# Crossroads between light response and nutrient signalling: ENV1 and PhLP1 act as mutual regulatory pair in *Trichoderma reesei*

**DOI:** 10.1186/1471-2164-15-425

**Published:** 2014-06-04

**Authors:** Doris Tisch, Andre Schuster, Monika Schmoll

**Affiliations:** Research Area of Gene Technology and Applied Biochemistry, Institute for Chemical Engineering, Vienna University of Technology, Gumpendorferstraße 1a, A-1060 Wien, Austria; AIT Austrian Institute of Technology, Department Health and Environment, Bioresources, Konrad Lorenz Strasse 24, 3430 Tulln, Austria

## Abstract

**Background:**

Crosstalk between the signalling pathways responding to light–dark cycles and those triggering the adaptation of metabolism to the environment is known to occur in various organisms. This interrelationship of light response and nutrient sigalling is crucial for health and fitness. The tropical ascomycete *Trichoderma reesei* (syn. *Hypocrea jecorina*) represents one of the most efficient plant cell wall degraders. Regulation of the enzymes required for this process is affected by nutritional signals as well as other environmental signals including light. Therefore we aimed to elucidate the interrelationship between nutrient and light signaling and how the light signal is transmitted to downstream pathways.

**Results:**

We found that the targets of the light regulatory protein ENV1 in light show considerable overlap with those of the heterotrimeric G-protein components PhLP1, GNB1 and GNG1. Detailed investigation of a regulatory interrelationship of these components with ENV1 under conditions of early and late light response indicated a transcriptional mutual regulation between PhLP1 and ENV1, which appears to dampen nutrient signalling during early light response, presumably to free resources for protective measures prior to adaptation of metabolism to light. Investigating the downstream part of the cascade we found support for the hypothesis that ENV1 is necessary for cAMP mediated regulation of a considerable part of the core functions of the output pathway of this cascade, including regulation of glycoside hydrolase genes and those involved in nitrogen, sulphur and amino acid metabolism.

**Conclusions:**

ENV1 and PhLP1 are mutual regulators connecting light signaling with nutrient signaling, with ENV1 triggering the output pathway by influencing cAMP levels.

**Electronic supplementary material:**

The online version of this article (doi:10.1186/1471-2164-15-425) contains supplementary material, which is available to authorized users.

## Background

The role of light in life has been subject to intensive research with almost all living organisms from animals to bacteria, plants and fungi. In recent years, interconnections of circadian rhythmicity and its resetting by light with regulation of metabolic pathways became obvious. Proper integration of adaptation to light–dark cycles and adequate adjustment of metabolism is known to be crucial for health, fitness and sometimes even survival of plants [1, 2], mammals [3, 4] and fungi [5]. However, although numerous genes are known to play a role in the integration of nutrient signalling with light dependent adjustment to the environment [6], the molecular basis for the underlying regulatory network remains to be explored in detail.

In fungi, the light status in the environment has a more general effect than only triggering sporulation, defence mechanisms or DNA-photolyases in fungi [5, 7]. Studies on fungi during the previous century until present time showed that light has an impact on nearly all metabolic processes in fungi like carotenoid metabolism, polysaccharide metabolism, fatty acid metabolism, nitrogen and sulphur metabolism and many more [8]. One of the first model organisms for investigations of light effects on morphology in fungi was *Trichoderma* [9], because of its visible reaction to illumination, which triggers conidiation [10, 11]. In the potent plant cell wall degrader *T. reesei* [12, 13] signalling events causing regulation of plant cell wall degrading enzymes as well as light response are subject to continued research efforts [9, 14, 15]. One of the most prominent and hence also best studied mechanisms for this purpose is the pathway of heterotrimeric G proteins. G proteins consist of alpha, beta and gamma subunits. The heterotrimeric complex receives a signal from the G protein coupled receptors (GPCR), which is influenced by external stimuli. In case of activation of the GPCR, a conformational change of the receptor results in an exchange of GDP for GTP at the G alpha subunit. The active G alpha subunit dissociates from the tightly bound G beta gamma dimer and both are impacting their effectors to affect regulatory pathways of secondary messengers [16, 17].

Among these pathways, the cAMP pathway represents an important output of heterotrimeric G-protein signaling. In this pathway, the intracellular levels of the secondary messenger cAMP is regulated by adenylate cyclase, which synthesizes cAMP and phosphodiesterase (PDE), which degrades cAMP [18]. Steady state levels of cAMP were found to be fine-tuned by a negative feedback loop established by PDE and protein kinase A (PKA), which is activated by cAMP [19–21].

In *T. reesei*, transcript levels of the genes encoding the G protein alpha subunits GNA1 and GNA3 are regulated by light and the light regulatory protein ENV1 [22–25]. ENV1 was found to be essential for elevated cAMP levels and is assumed to negatively influence phosphodiesterase activity [25]. The cAMP pathway as a main output pathway of heterotrimeric G-protein signalling was shown to be important for cellulase gene expression in *T. reesei* [26, 27]. ENV1 is a PAS/LOV domain containing regulator of light responses and cellulase gene expression [22, 28, 29]. Its closest characterized homologue is the *Neurospora crassa* photoreceptor protein VVD, which plays a role in regulation of the circadian rhythm, photoadaptation and sensing of light intensities [30–32]. VVD is also known to act as a universal brake in light responses [33] and to modulate activity of the White Collar Complex (WCC) by physical interaction [34, 35].

Transcription of *env1* is strongly induced by light and this induction is dependent on the blue light photoreceptors BLR1 and BLR2 [28]. Interestingly, ENVOY also possesses regulatory functions in darkness [29] and represents a crucial signalling component with functions not only in light response, but also in nutrient signal transduction [15].

The investigation of a potential link between light signalling and the pathway of heterotrimeric G proteins revealed the class I phosducin like protein PhLP1 as a central component. Additionally, the G protein beta and gamma subunits GNB1 and GNG1 were found to be members of this regulatory mechanism, all of which are crucial for tight regulation of light response in *T. reesei* [14]. Transcription of *phlp1* is responsive to light, with *phlp1* clearly belonging to the late light responsive genes (LLRGs) as defined by Chen [33]. Microarray analysis of mutants lacking PhLP1, GNB1 or GNG1 showed that their primary function is a positive regulation of target genes in light, with glycoside hydrolases as an important output pathway. These findings support the idea of a connection between nutrient and light signalling via heterotrimeric G-protein signalling [14]. In agreement with this finding a study in *Trichoderma atroviride* showed that the photoceptors BLR1 and BLR2 are crucial for the light stimulated nutrient uptake [36].

Based on the extensive evidence for an interconnection between nutrient signalling and light response, we now tackled the issue how this regulatory interaction is established at the molecular level and how the signal is transmitted further. To this end we investigated the first step of regulation by adjustment of transcript levels, that represents the basis for translation, modification and ultimately signaling output. We compared genome wide transcriptional regulation by ENV1 with that of the heterotrimeric G-protein components GNB1, GNG1 and PhLP1, which pointed at a mechanism coupling the light signal with the G protein pathway and with glycoside hydrolases as representatives of the nutrient degradation machinery as output pathway. Our subsequent analyses of light response of selected signalling components in numerous mutant strains revealed that mutual regulation of ENV1 and PhLP1 constitutes one node in the interconnection between nutrient and light signalling, with GNB1 as an important factor of signal transmission to downstream targets. Subsequently, we show that the core output functions impacted by ENV1 are regulated via its effect on cAMP levels.

## Results

### Targets of light- and nutrient signalling show considerable correlation

In order to evaluate the interrelationship between nutrient and light signaling we compared the regulatory targets of these pathways as revealed by transcriptome analysis from strains grown with microcrystalline cellulose as sole carbon source in light and darkness. Thereby, ENV1, BLR1 and BLR2 [15] served as representatives of the light response pathway and PhLP1, GNB1 and GNG1 [14] represented the nutrient signaling pathway of heterotrimeric G-proteins.

Interestingly, our analysis of the influence of the light response machinery on gene regulation in light and darkness had revealed the strongest effect on positive targets of ENV1, BLR1 and BLR2 in light (i.e. underexpression of genes in the respective mutants in LL compared to the parental strain), the most severe influence being exerted by ENV1 [15]. This condition is similar to the condition most relevant for the function of PhLP1, GNB1 and GNG1 [14]. Because of the outstanding position of ENV1 in positive regulation of downstream targets in light, we compared the positive PhLP1-GNB1-GNG1 targets [14] with those of ENV1 in light (Additional file 1, Dataset 1).

Intriguingly, we found 77% (483 genes) of the positive targets of PhLP1-GNB1-GNG1 to overlap with those of ENV1 in light. In principle, the detected target processes strongly resemble those of the light signalling machinery. Gene set enrichment analysis of these common targets with the p-value threshold for significant enrichment set to ≤0.005 revealed enrichment in genes involved in metabolic processes, transport, oxidoreductase activity and regulation. A specific enrichment of polygalacturonase activity, mainly represented by genes encoding glycoside hydrolases of family 28, suggests that one common target of ENV1, PhLP1, GNB1 and GNG1 could be the enhancement of maceration and soft rotting of plant tissue by weakening the pectin network.

We conclude that the nutrient signals transmitted via PhLP1-GNB1-GNG1 are closely interrelated with light signalling via ENV1. Lack of one of these four components presumably causes the system mediating the respective positive signalling output to shut down. The data confirm the key function of ENV1 and PhLP1-GNB1-GNG1 in interconnecting nutrient- and light signalling.

### Light is the most important source of variation in target genes

**Figure 1:**
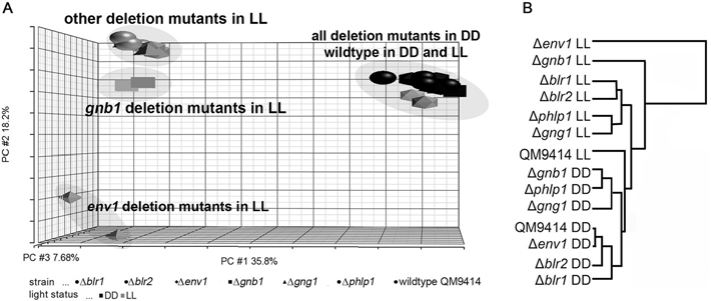
**Analysis of global genome wide transcript abundance patterns of Δ** ***blr1*** **, Δ** ***blr2*** **, Δ** ***env1,*** **Δ** ***phlp1*** **, Δ** ***gnb1*** **and Δ** ***gng1*** **by (A) Principal Component Analysis (PCA) and (B) hierarchical cluster analysis** ***.*** Data on transcript levels of all 9127 genes of *T. reesei*[14, 15] in the parental strain as well as mutant strains in light and darkness of two biological replicates were used for the analysis. Average standard deviation between two biological replicates was 13%.

In order to evaluate the interconnection between light and nutrient signal transmission (in terms of regulatory targets), we performed a hierarchical cluster analysis and principal component analysis (PCA) of genome wide transcript patterns of all strains included in this study (Figure 1). Except for the wildtype, the detected clusters revealed a clear separation between gene regulation in strains grown in light compared to those grown in darkness. This finding suggests that the tight regulation of light response in the wildtype becomes unbalanced due to the lack of either a central component of the light signalling machinery or a component involved in light dependent transmission of nutrient signals.

However, Δ*env1* in light appears in this cluster as an outgroup, hence highlighting its distinct function, and Δ*gnb1* in light also occupies a peculiar position. Nevertheless, nutrient and light- signalling strains still appear in separate clusters according to cultivation in light and darkness. The outstanding position of Δ*gnb1* in light supports the hypothesis that PhLP1 and GNG1 are important for appropriate folding of GNB1 in light [14], the function of which obviously is more different from that of PhLP1 and GNG1 in light than in darkness (where deletion of any of the three genes has rather similar consequences). PCA analysis confirms this result and shows that light is the major source of variation among the different strains (Figure 1). We conclude that both ENV1 and GNB1 have crucial functions in regulation of downstream output pathways, with ENV1 having an even more widespread effect than GNB1.

### Evaluation of the interconnection between light- and nutrient signalling

**Figure 2:**
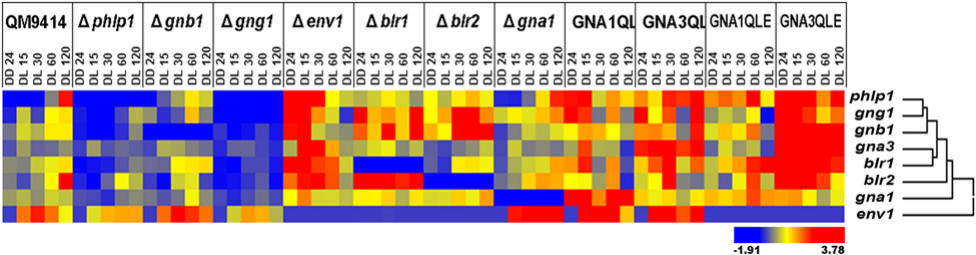
**Overview on qRT PCR analysis of** ***blr1*** **,** ***blr2*** **,** ***env1*** **,** ***gnb1*** **,** ***gng1*** **,** ***phlp1*** **,** ***gna1*** **and** ***gna3*** **in strains with alterations of these genes.** Hierarchical cluster analysis was performed to evaluate similar regulation patterns of genes in the different mutants. Data for the strains GNA1QLE and GNA3QLE, which express constitutively activated versions of the respective G-protein alpha subunits and lack the *env1* open reading frame were taken from [25] in order to provide a complete picture.

Because of the considerable overlap between targets of the light and nutrient signalling pathway via ENV and PhLP1-GNB1-GNG1, we were interested how the interconnection between the two pathways is established. Since an influence of ENV1 on the genes encoding the G protein alpha subunits GNA1 and GNA3 is known [25], we included these G protein alpha subunits in the analysis along with double mutants bearing constitutive alleles and a deletion in *env1*. qRT-PCR analysis of gene transcription in the parental strain QM9414 and strains altered in components of the signal transduction pathways of light response and heterotrimeric G protein signalling were applied to reveal the hierarchical order and interrelationships of regulators within the cascade. In order to minimize influences of growth defects or altered expression of hydrolytic enzymes in light, we chose glycerol as carbon source. Glycerol does not induce cellulase gene expression in *T. reesei*, but does not prevent induction of cellulase gene expression upon addition of an inducer [37]. Additionally, we wanted to gain information on transient processes in addition to constant light and and therefore applied a switch from constant darkness to constant light after 24 hours of growth. This experimental design also enabled us to distinguish regulatory impacts on early and late light response: strains were incubated in darkness first and illuminated for 15, 30, 60 and 120 minutes. Two biological replicates were evaluated throughout the analysis. With this analysis we also aimed to identify the central component(s), from which the integrated signal is channelled for adjustment of the output pathways. Figure 2 provides an overview on results of qRT-PCR, which will be discussed in detail in the following.

### ENV1 acts negatively on transcription of photoreceptors

**Figure 3:**
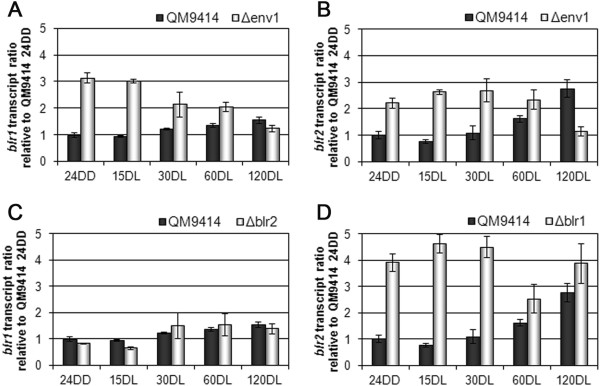
**Analysis of** ***blr1*** **and** ***blr2*** **transcript levels.** Transcript abundance is shown in response to light by qRT-PCR in the wildtype and ∆*env1* strain (**A** and **B**) as well as in the ∆*blr1* and ∆*blr2* strains (**C** and **D**). The strains were cultivated on Mandels-Andreotti minimal media with 1% (w/v) glycerol as carbon source. The strains were kept in darkness for 24 hours and then exposed to light. Samples were taken in darkness, indicated as 24DD, and after 15, 30, 60 and 120 minutes of illumination (displayed as minutes DL), respectively.

We first tested the regulatory connections of the photoreceptor genes. *env1* transcription rapidly reacts to light [22] and hence represents an ELRG. It was already shown that in the absence of either BLR1 or BLR2, transcription of *env1* is not induced and remains at barely detectable dark-levels [28]. Our analysis showed that ENV1 in turn influences photoreceptor transcript levels negatively in darkness and early light response, when *env1* is strongly induced in the parental strain (Figures 3A and B). Additionally, also *blr1* and *blr2* are not transcribed independently under transient conditions of light response, since BLR1 has a clearly negative effect on *blr2* transcription (Figures 3C and D). Consequently, we propose a model in which after induction of ENV1 expression, its negative effect on *blr1* and *blr2* transcription leads to a steady state level of transcription of these three genes. The repressing effect of BLR1 on *blr2* transcript levels is in accordance with earlier data in *T. atroviride* showing that BLR2 is the limiting factor for photoperception and phototransduction [38].

### Availability of PhLP1 has a positive effect on transcript levels of *gnb1*and *gna1*

**Figure 4:**
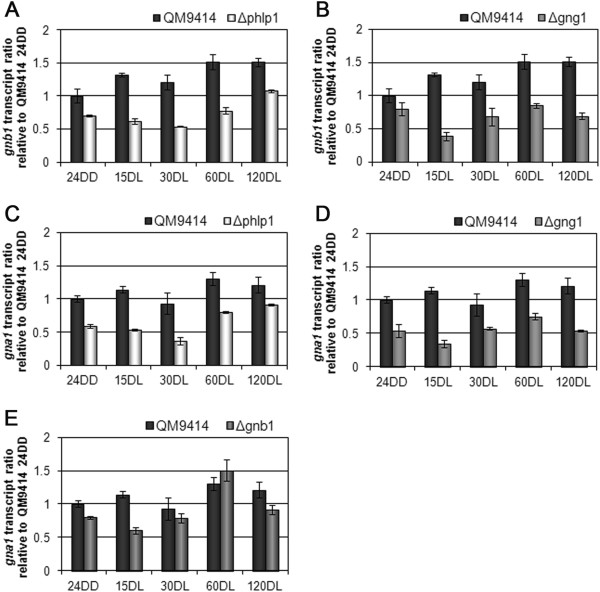
**Regulation by PhLP1, GNB1 and GNG1.** Influence on *gnb1* (**A** and **B**) and *gna1* (**C** and **D**) transcript levels in response to light and influence of GNB1 on *gna1* transcription **(E)**. Transcript levels were measured by qRT-PCR from strains grown on Mandels-Andreotti minimal media with 1% (w/v) glycerol as carbon source. Strains were grown on Mandels-Andreotti minimal media with 1% (w/v) glycerol as carbon source for 24 hours in darkness (24DD) and then exposed to light for 15, 30, 60 or 120 minutes (minutes DL), respectively.

Class I phosducin like proteins are supposed to act as co-chaperones for G protein beta and gamma folding [39]. Assuming that also in our system a tight regulation of the amount of functional G protein subunits occurs, we investigated whether the availability of PhLP1 and hence efficiently folded GNB1 and GNG1 would feed back to the respective transcript levels. We found that presence of PhLP1 enhances *gnb1* transcript levels (Figure 4A). Transcript levels of *gnb1* in Δ*gng1* also showed a positive effect of GNG1 on *gnb1* transcription (Figure 4B), hence suggesting that both PhLP1 and GNG1 are important for regulation of GNB1. The amount of the resulting functional G protein beta-gamma complex would consequently be determined by upregulation of GNB1 in this three way regulatory interaction. This result is in agreement with data from constant illumination and on cellulose [14], which indicates the central function of GNB1 to be carbon source independent. Moreover, the function of GNB1 is concluded to extend beyond a transient effect after illumination.

While the effect of PhLP1, GNB1 or GNG1 on transcription of *gna3* was negligible (data not shown), we found a clearly positive influence of PhLP1 and GNG1 on transcription of *gna1* (Figures 4C and D). Interestingly, as seen for the effect of these two factors on *gnb1*, also their effect on transcription of *gna1* was similar. Considering that class I phosducin like proteins are assumed to act as co-chaperones, these data can be interpreted in a way that lack of either of these genes causes folding and complex formation with GNB1 to fail, which may also be responsible for the effect on *gna1*. However, no absolute positive effect of GNB1 on the *gna1* transcript pattern was observed (Figure 4E).

### The G protein alpha subunits act on transcription of *phlp1*but not *gnb1*or *gng1*

**Figure 5:**
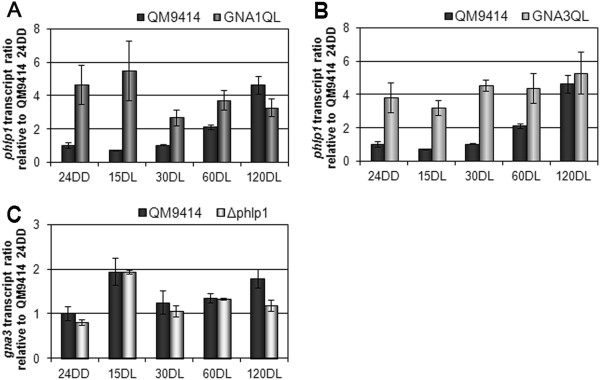
**Effect of constitutive activation of GNA1 or GNA3 and impact of** ***phlp1*** **.** Transcript ratios of *phlp1* (**A** and **B**) and the G protein alpha subunit gene *gna3* **(C)** in response to light upon growth on glycerol as carbon source are shown. qRT-PCR measurements show that both constitutively activated G alpha subunits (GNA1QL and GNA3QL) cause enhanced *phlp1* transcript levels (**A** and **B**). PhLP1 positively influences *gna1* transcript levels (Figure 4C), but not *gna3* transcript levels **(C)**. Strains were grown on Mandels-Andreotti minimal media with 1% (w/v) glycerol as carbon source for 24 hours in darkness (24DD) and then exposed to light for 15, 30, 60 or 120 minutes (minutes DL), respectively.

In order to establish the position of the G protein alpha subunits GNA1 and GNA3 within the network, we investigated transcription patterns of their genes in mutants bearing constitutively activated versions of these genes (GNA1QL and GNA3QL; [23, 24]). Interestingly, we found a positive interconnection of *gna1* with *phlp1*, since they showed a mutually positive effect on each other’s transcription in early light response (Figures 5A and C). Similarly, also GNA3 acts positively on transcription of *phlp1* (Figure 5C), although no effect of PhLP1 on *gna3* was observed (Figure 5D). In contrast, no significant impact of GNA1 or GNA3 on transcription of the beta- and gamma subunit genes *gnb1* and *gng1* was observed, hence placing PhLP1 in a crucial position in the nutrient signalling cascade, likely as signal transmitter towards the output pathway, but due to its regulatory interaction with *gna1* and *gnb1* also as part of a feedback cycle.

### PhLP1 and ENV1 are regulatorily interconnected

**Figure 6:**
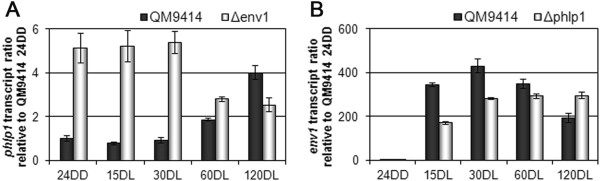
**Regulatory interrelationship of PhLP1 and ENV1 at the level of transcription. (A)** *phlp1* transcript levels are enhanced in ∆*env1* mutant strain compared to the wildtype. **(B)** *env1* transcript levels are decreased in the ∆*phlp1* mutant strain compared to the wildtype. Strains were cultivated on glycerol as carbon source for 24 hours in darkness (24DD) and then exposed to light for 15, 30, 60 or 120 minutes (minutes DL), respectively.

With *phlp1* emerging as a potential node between nutrient signalling and light response, we now analysed at which part of the light response pathway *phlp1* might intervene. Recently it was shown that the photoreceptors BLR1 and BLR2 only have marginal influence on *phlp1* transcription. In contrast, ENV1 reduces the differential transcription of *phlp1* in light and darkness and seems to be more relevant for transcriptional regulation of *phlp1* than BLR1 or BLR2 [14]. Therefore, we were interested in the mechanistic roles of PhLP1 and ENV1 under conditions reflecting early and late light response. We found that ENV1 has a clearly negative effect on transcription of *phlp1* and on the other hand PhLP1 acts positively on early light response of *env1* transcription (Figures 6A and B). Hence, these two components are likely to establish a steady state level by positive and negative (indirect) transcriptional interaction upon illumination.

**Figure 7:**
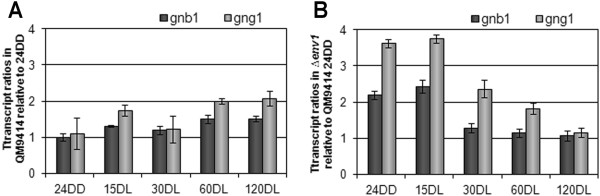
**Regulatory interrelationship of GNB1, GNG1 and ENV1 at the level of transcription. (A)** *gnb1* and *gng1* show similar transcript patterns in the wildtype strain. **(B)** *gnb1* and *gng1* transcript patterns in ∆*env1* show an upregulation at early time points. Strains were cultivated on glycerol as carbon source for 24 hours in darkness (24DD) and then exposed to light for 15, 30, 60 or 120 minutes (minutes DL), respectively.

Since PhLP1 impacts transcription of the G protein beta subunit gene *gnb1*, we were interested whether ENV1 would also influence *gnb1*. Indeed we found that ENV1 negatively regulates transcription of both *gnb1* and *gng1*, the transcription patterns of both genes being highly similar in the Δ*env1* mutant (Figures 7A and B). Notably, the regulatory interaction between ENV1 and PhLP1 as well as the influence of ENV1 on transcription of *gnb1* and *gng1* are most obvious during early stages of light response. In contrast, the effect of PhLP1 and GNG1 on transcription of *gnb1* can be detected also after early light response. We conclude that the influence of ENV1 on transcript levels of *gnb1* and *gng1* is likely to be mediated via its negative effect on PhLP1.

### Regulation of the output pathways by cAMP levels

The cAMP pathway is considered the major output pathway of heterotrimeric G-protein signaling [40] and would hence be likely to act as a coincidence detector between nutrient and light signaling. For *T. reesei*, an influence of the two heterotrimeric G-protein alpha subunits GNA1 and GNA3 on intracellular cAMP levels was observed [23, 24]. Having established the node between nutrient and light signalling we now wanted to investigate the downstream pathway of this cascade. Deletion of *env1* is known to result in strongly decreased intracellular cAMP levels [25], which are likely to represent an output of the regulatory interconnection between light- and nutrient signalling established between *env1* and *phlp1*. In the strain lacking *acy1*, cAMP production is abolished [26] and consequently the situation resembles that in the strain lacking *env1*.

Hence, we performed microarray analysis of a deletion mutant in the gene encoding adenylate cyclase 1 (*acy1*), which synthesizes cAMP. Similar conditions to those used for previous transcriptome analysis of ENV1 [15] i. e. growth on cellulose for 72 hours in light and darkness were applied. We compared the results with the transcriptome of Δ*env1* in light and darkness. Indeed, we found a considerable overlap of targets, interestingly only among targets in light and no overlapping targets were found in darkness (Additional file 2, Dataset 2). The 31 genes up-regulated in light in Δ*env1* and Δ*acy1* included one polyketide synthase (TR_65891), three putative G-protein coupled receptors (TR_109146, TR_103694 and TR_72627) and one transcription factor related to a *N. crassa* transcription factor responsive to light (TR_72057), as well as two sugar transporters (TR_65153 and TR_82309). 114 genes were underexpressed in light in Δ*env1* and Δ*acy1*, among them 25 glycoside hydrolases including *xyn2*, *xyn4*, *bxl1*, *cel3a*, *cel3b*, *egl1*, *egl2*, *egl6*, *cbh2*, *cel61a* and *cel61b* as well as the genes encoding the auxiliary proteins swollenin, CIP1 and CIP2 and the cellulase and hemicellulase regulator gene *xyr1*. Additionally, we found 16 genes involved in sulphur metabolism, including the regulator gene *lim1* [41]. Further genes consistently regulated in Δ*env1* and Δ*acy1* include two G-protein coupled receptors (TR_121990 and TR_53238) as well as six putative sugar transporters including the recently characterized lactose permease TR_3405, which is also important for cellulase gene expression on lactose [42]. No contrasting regulation was observed (overexpression in Δ*env1* but underexpression in Δ*acy1* or *vice versa*).

Functional category analysis revealed that the functions of the consistently regulated genes are enriched in metabolism (p-value 1.91 e-12), especially amino acid metabolism (p-value 1,58 e-06), nitrogen and sulphur metabolism (p-value 1.33 e-11) as well as C-compound and carbohydrate metabolism (p-value 3.89 e-09). In the latter group particular enrichment in polysaccharide metabolism (p-value 4.78 e-12) was observed. Additionally, transport functions of carbohydrates and amino acids were enriched. We conclude that a considerable portion of the core functions of ENV1 (regulation of glycoside hydrolases, sulphur metabolism and transport) are regulated via the effect of ENV1 on cAMP levels.

## Discussion

**Figure 8:**
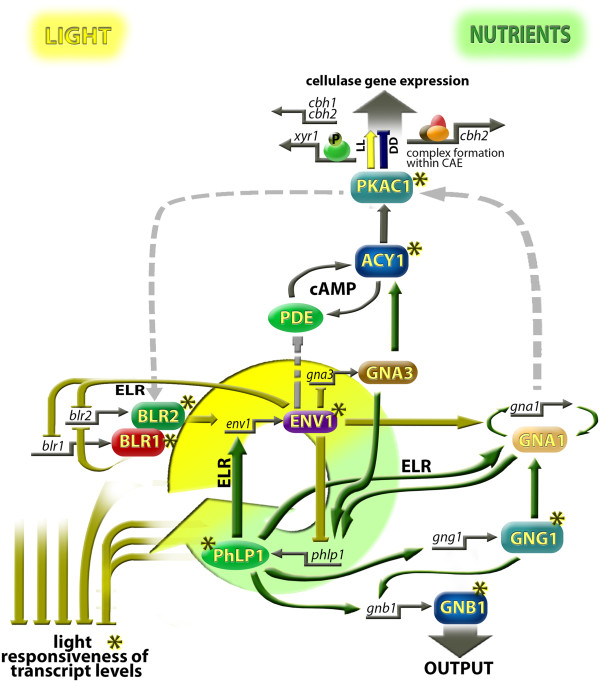
**Schematic drawing of the current model for the interrelationship between nutrient and light signaling in** ***T. reesei*** **.** The regulatory interplay between nutrient and light signalling involves a limb represented by components of the heterotrimeric G-protein pathway and a limb established by the crucial components of the light response machinery. Central interactors of this connection are ENV1 and PhLP1. The positive effect of PhLP1 on *env1* transcription in early light regulation (ELR) may be responsible for negative regulation of heterotrimeric G-protein signalling by ENV1. The effect of ENV1 on *gnb1* and *gng1* is mediated by PhLP1. Investigation of the effect of constitutive activation of the G-alpha subunits GNA1 and GNA3 on regulation of *phlp1* transcript levels showed that the nutrient signals transmitted by GNA1 and GNA3 impact *phlp1*. Both the light signalling components as well as the factors of heterotrimeric G-protein pathway analyzed in this study were found to dampen light responsiveness of transcript abundance and are likely to constitute a light specific regulatory mechanism sustaining transcript levels of downstream genes relevant during growth in light. Investigation of the cAMP pathway as depicted in this scheme was described earlier [26]. Arrows indicate positive influence while plungers indicate a negative effect. Dashed lines show hypotheses deduced from results in other fungi (discussed in [26]). Asterisks indicate an effect on light responsiveness of transcript levels.

The major influence of light on fungi was studied extensively in the last decades and it could be shown that light affects a broad spectrum of metabolic pathways, morphological changes, growth and secondary metabolism [7, 8] . Since the discovery, that cellulase gene expression is modulated by light [22], studies unravelling the light signalling network influencing the cellulase gene expression were undertaken [14, 15, 23–25, 28, 29, 43], which revealed a puzzle of components regulating the model output pathway of cellulase transcription in response to light. Besides the intriguing insights into fungal physiology, these findings also provide perspectives for research towards elucidation of the interplay between light response and metabolism in higher organisms. The results presented here connect these components by placing them in the background of a signaling network and provide intriguingly new insights into the mechanism how light- and nutrient signalling are connected at the molecular level (Figure 8).

Together with previous studies, the model we propose illustrates the predominantly negative impact of the light signalling machinery and in contrast a largely positive effect of PhLP1 and other components of heterotrimeric G-protein signalling on regulation of nutrient utilization or enzyme gene transcription, respectively. Hence, the interplay of a positive and a negative limb are proposed to establish a balanced output which integrates both light and nutrient signals. This mechanism is reminiscent of the positive and negative regulatory cycles triggering light response and circadian rhythmicity in *N. crassa* [44]. The dissimilarity of the *gnb1* transcript pattern from those of *phlp1* and *gng1* suggests that lack of PhLP1 and GNG1 has different consequences than lack of *gnb1*. Hence the alterations seen cannot be exclusively due to perturbed complex formation of GNB1 and GNG1 as supported by PhLP1. In mammalian systems individual functions of G-beta subunits independent of the G-gamma subunit were reported [45], which may be one reason for this phenomenon. This hypothesis is supported by the finding that lack of GNB1 causes a slightly different phenotype than that of PhLP1 or GNG1 [14] and the characteristic alterations in transcriptome data for a strain lacking GNB1, reflecting partially different targets.

It has to be considered, that the regulatory interconnections revealed in this study must be mediated by transcription factors, which remain to be determined and might include the photoreceptors BLR1 and BLR2. Due to the observed function of ENV1 via adjustment of cAMP levels it appears likely that this regulation also involves posttranslational modification and hence activation/deactivation of these transcription factors by phosphorylation. These transcription factors could either act directly on the target promotors or alternatively activate/deactivate further regulators. The latter hypothesis would be supported by the finding that in *N. crassa* regulation by WC-1 is mediated by a flat hierarchical transcription factor network [46]. Analysis of interaction partners of the signaling components investigated in this study will provide insight into the mechanisms involved in this regulation and is currently in progress in our lab.

Transcriptome analysis revealed a crucial position of ENV1 and GNB1 in the interrelationship of light response and nutrient signalling in *T. reesei*. Interestingly, upon deletion of the respective genes (*env1* and *gnb1*), we saw a considerable increase in genes regulated by light from roughly 3% to more than 23 or 30%, respectively [14, 15]. The crucial importance of ENV1 in both nutrient and light signalling is further reflected by the considerable number of regulatory targets distinct from those of the photoreceptor proteins [15]. Most of the genes differentially regulated in Δ*env1* or Δ*gnb1* were downregulated in light, although in Δ*env1* considerable upregulation also occurs. The huge impact of light on strains lacking *env1* was already observed in earlier studies [22, 25, 28, 47], but the extent of the regulatory function of ENV1 in light was still unexpected. Also in strains lacking BLR1 or BLR2 considerable downregulation of transcript levels occurred [15]. These findings led us to hypothesize that the light response machinery and particularly ENV1 sustains expression levels of genes responsible for environmental sensing and signal transmission in light, presumably also via PhLP1-GNB1-GNG1. As these components appear to only have few targets in darkness, this mechanism might not be operative in darkness, which raises the question whether a different pathway is responsible for such an effect in darkness. Correlation of the majority of targets of PhLP1-GNB1-GNG1 with those of ENV1 along with the peculiar position of Δ*env1* in the cluster analysis of mutant strains and its impact on regulation of G-alpha subunit genes as described earlier [25], places ENV1 upstream of the components of heterotrimeric G protein signalling. Nevertheless, our new transcriptome data on Δ*acy1* also indicate that ENV1 plays an important role in the output pathway of the cascade and that the components of heterotrimeric G-protein signalling analysed here might just be a modulator of the pathway regulated by ENV1. Future research will show if this hypothesis can be substantiated.

Evaluation of genome wide transcription patterns and subsequent detailed analysis of light response in several mutant strains enabled us to identify ENV1 and PhLP1 as a central interlocked regulatory pair connecting the light response pathway with nutrient signalling. Thereby, PhlP1 acts positively on *env1* transcription during early light response, which in turn has a negative effect on transcript abundance of *blr1* and *blr2* at this time. Considering data on the effect of VVD on the WCC [35], ENV1 likely also supports inhibition of BLR complex activity. ENV1 in turn consistently acts negatively on transcript levels of not only *phlp1*, but also *gnb1* and *gng1*. This result is in accordance with earlier findings in *N. crassa* [33], showing that early light-responsive genes are mostly involved in primary measures to adjust to the light conditions, including protection from light, photoperception and cell signalling, while functions in C-compound and carbohydrate metabolism predominate among late light responsive genes. Therefore our findings could be interpreted in a way that ENV1 dampens G-protein signalling during early light response by decreasing transcription of *phlp1*, *gnb1* and *gng1* in order to provide resources for protective measures. The initial positive action of PhLP1 on *env1* transcription enhances this effect. Subsequently, the positive action of PhLP1 on complex formation of the G beta and gamma subunits and hence G-protein signalling – which is likely to transmit nutritional signals - might be important for metabolic adaptation to light. In the context of the overlap of transcriptomes of deletion mutants in *env1*, *phlp1*, *gnb1* and *gng1* it becomes clear that this interrelationship is even more profound and extends beyond transient effects in early light response.

As for the downstream pathway of the node between PhLP1 and ENV1, we investigated to which extent the regulation of cAMP levels by ENV1, which is assumed to be accomplished by a negative effect on phosphodiesterase activity [25], is relevant for gene regulation by ENV1. As deletion of adenylate cyclase has a similar effect on cAMP levels as deletion of ENV1 (despite different mechanisms), we performed microarray analysis of Δ*acy1* in light and darkness on cellulose for identification of cAMP dependent targets of ENV1. Interestingly, we only detected an overlap of regulated genes in light, with most of the consistently regulated genes being upregulated. The functions of these genes are strongly enriched in metabolic functions – specifically carbon and sulphur/amino acid metabolism. Interestingly, the sulphur source in the medium is crucial for the ability of *T. reesei* to utilize cellulose. Without sulphate, growth of *T. reesei* on cellulose in light is severely perturbed and methionine cannot serve to replace sulphate as sulphur source under these conditions [41]. Our findings hence indicate that the interrelationship between sulphur and carbon metabolism is likely to involve the function of cAMP dependent mechanisms. This overlap in regulated genes in light is further in agreement with the retarded growth phenotype of *T. reesei* strains lacking *env1* [22, 47], as retarded growth is also observed upon lack of *acy1* [26]. However, the mechanism for regulation of growth by cAMP levels in *T. reesei* remains to be investigated in detail.

Knowledge on the overlap of genes regulated as a consequence of two different factors crucial for normal growth of *T. reesei* should enable insight in to the mechanism regulating growth in response to cAMP levels. In order to narrow down the number of candidate regulators, we checked whether *N. crassa* homologues of the target genes of ENV1 and ACY1 in light, for which phenotype analyses of mutants are available, are known to be important for normal growth. Abnormal growth patterns in knock outs were only found for homologues of TR_54675 (NCU03725; *vib-1*) and TR_56952 (NCU05990; putative cell surface receptor/MFS transporter). Transcript levels of TR_56952 in Δ*acy1* and Δ*env1* are only downregulated about 2fold. Therefore, we consider it unlikely that this putative transporter might contribute to the severe growth defect of Δ*acy1* and Δ*env1. N. crassa* VIB-1 is among other functions in cell recognition and programmed cell death, important for response to carbon starvation [48]. The transcript abundance of TR_54675 is decreased 3.7fold in Δ*acy1* and more than 19fold in Δ*env1*. However, the *vib-1* deletion mutant only shows a decreased linear growth of about 20% compared to wild-type. Consequently, TR_54675 may be in part responsible for the retarded growth in response to reduced cAMP levels, but additional factors with more significant effects remain to be identified.

## Conclusions

In summary, we found that a sizable amount of independent targets of ENV1 in light shows considerable overlap with targets of the heterotrimeric G-protein components PhLP1, GNB1 and GNG1. Complementary investigation of early and late light response revealed that ENV1 represents a crucial node in light signal transduction and exerts its function in part via the regulatory interrelationship with the phosducin like protein PhLP1, as well as GNB1 and GNG1. This interaction between nutrient and light signalling is at least in part mediated by transcriptional interaction of ENV1 and PhLP1. Downstream targets of the cascade are to a considerable extent regulated via the function of ENV1 in modulation of cAMP levels.

## Methods

### Strains and culture conditions

As parental strain *Trichoderma reesei* QM9414 (ATCC 26921) was used. Additionally the recombinant strains ∆*env1*, ∆*blr1* and ∆*blr2* [28], ∆*gna1,* GNA1QL [24], GNA3QL [23], ∆*phlp1*, ∆*gnb1*, ∆*gng1* [14] and ∆*acy1* [26] were analysed throughout this study.

Strains were cultivated in 1 L shake flasks at 28°C on a rotary shaker (200 rpm) on Mandels-Andreotti minimal medium [49], supplemented with 0.1% (w/v) peptone to induce germination and with 1% (w/v) carbon source. For transcriptome analysis of ∆*acy1* we used microcrystalline cellulose as carbon source. ∆*acy1* was grown in constant light (LL; 1500 lux) or constant darkness (DD) for 72 hours in order to correspond to the conditions used previously [14, 15]. For light response experiments, glycerol (Merck, Darmstadt, Germany) was used as sole carbon source, Strains were kept in constant darkness for 24 hours (24DD) and were exposed to light thereafter (DL; 1500 lux) as indicated with the respective figures. Harvesting of dark grown cultures was performed under safe-red-light (darkroom lamp, Philips PF712E, red, E27, 15 W).

### Nucleic acid isolation and manipulation

Mycelium for isolation of nucleic acids was harvested from flasks by filtration, briefly rinsed with tap water and snap-frozen in liquid nitrogen. Isolation of total RNA was done as described elsewhere [25]. RNA concentration was analysed using a Nanodrop ND-1000 spectrophotometer (PEQLAB, Erlangen, Germany). Total RNA was treated with DNase I (Thermo Fisher Scientific, Vienna, Austria) and the RNeasy Plant Mini Kit (QIAGEN, Hilden, Germany) was used for purification. Quality control of total RNA was performed using the Experion Automated Electrophoresis System (Bio-Rad, Hercules, USA) and the Experion RNA StdSens Analysis Kit (Bio-Rad). The threshold for minimum quality for use in our experiments was set to RQI > 7, although the majority of our samples had RQI factors of >9.

### Quanitative reverse transcription PCR and microarray analysis

For microarray experiments, cDNA was prepared by reverse-transcription of five μg of purified total RNA using the RevertAid-H^−^ First Strand cDNA Synthesis Kit (Thermo Fisher Scientific) and Random Hexamer Primers following the manufacturer’s instructions. For cDNA to be analyzed by qRT-PCR we used oligo-d(T)-primers instead of the Random Hexamer Primers. IQ5 Icycler system (Bio-rad) in combination with the iQ SYBR Green supermix kit (Bio-rad) was used for qRT-PCR. For subsequent data analysis the REST software was applied [50]. Technical triplicates from at least two independent biological replicates were used for statistical calculations. The gene *rpl6e* encoding a ribosome subunit was tested for constitutive transcript levels in light and darkness and under different nutritional conditions [14, 25] and was therefore used as a reference gene for qRT-PCR assays (for primer sequences of all assays see Table 1).

**Table 1:** **Oligonucleotide sequences of primers used in this study**

Gene	Fragment	Reference	Sequence
*env1*	RTenv1F	This study	5‘ CATTGACCTTGGCCCTCTC 3’
*env1*	RTenv1R	This study	5’ GACAGTTTCGACCCATGATCTC 3’
*gna1*	RTgna1F	This study	5’ CACCACCATCCTCTTCCTG 3’
*gna1*	RTgna1R	This study	5’ CGTCTTGATGAACCACCTG 3’
*gna3*	RTgna3F	This study	5’ CTCACACAAGCCACCGACAC 3’
*gna3*	RTgna3R	This study	5’ ATGCCCGAATCCTTGAGC 3’
*blr1*	RTblr1F	This study	5’ CTTATACCTTTCGCCCTCGTG 3’
*blr1*	RTblr1R	This study	5’ GCCCGTTGTTGCGTCTTTC 3’
*blr2*	RTblr2F	This study	5’ ATCGCATGAGGAAGAAGGAC 3’
*blr2*	RTblr2R	This study	5’ GGGCGATTGGTTATTTGG 3’
*cbh1*	RTcbh1F	Tisch et al. [14]	5' ACCGTTGTCACCCAGTTCG 3'
*cbh1*	RTcbh1R	Tisch et al. [14]	5' ATCGTTGAGCTCGTTGCCAG 3'
*phd2*	RTphd2F	Tisch et al. [14]	5' GACAGGAGCTCGAGAAGGAAG 3'
*phd2*	RTphd2R	Tisch et al. [14]	5' CAAAGACGGCAACGGTAGTG 3'
*gnb1*	RTgnb1F	Tisch et al. [14]	5' CATCAACGACCGAAGCATC 3'
*gnb1*	RTgnb1R	Tisch et al. [14]	5' GCAGGCACCAGAAATGAAG 3'
*gng1*	RTgng1F	Tisch et al. [14]	5' CGTACTGCAATGGCACAAGAG 3'
*gng1*	RTgng1R	Tisch et al. [14]	5' GGATTGCTGAGGCGCATAG 3'
*rpl6e*	RTL6eF1	Tisch et al. [14]	5' GATACGTCATCGCCACCTCC 3'
*rpl6e*	RTL6eR1	Tisch et al. [14]	5' CTTCTCCTTGGCCTTCTCG 3'

The datasets used for this study are available under Gene Expression Omnibus (NCBI GEOdatasets), accession number GSE36448 for transcriptome data of photoreceptor strains [15]. Those from mutants in GNB1, GNG1 and PhLP1 [14] can be found under GSE27581 and data from transcriptome analysis of Δ*acy1* under GSE53874.

Data analysis for microarrays, principal component analysis (PCA) and gene set enrichment analysis (GSEA) was performed using PARTEK Genomics Suite 6.5 (PARTEK Inc., St. Louis, Missouri, USA), which applies ANOVA for evaluation of statistically significant differentially expressed genes. For hierarchical clustering the open source software HCE 3.5 was used with default settings [51]; http://www.cs.umd.edu/hcil/hce).

Datasets were evaluated using the community annotation including GO (Gene Ontology) classifications from the *T. reesei* genome database v2.0 provided by JGI (http://genome.jgi-psf.org/Trire2/Trire2.home.html) with revised annotations from [14].

## Availability of supporting data

The data sets supporting the results of this article are included within the article and its additional files at http://www.biomedcentral.com/1471-2164/15/425/abstract

## Electronic supplementary material

Additional file 1: **Dataset 2, Overlap of target genes of ENV1 with those of PHLP1, GNB1 and GNG1.** Genes at least twofold up- or downregulated compared to the parental strain upon growth on cellulose. (XLS 163 KB)

Additional file 2: **Dataset 2, Consistent gene regulation in mutants lacking** ***env1*** **or** ***acy1.*** Genes at least twofold up- or downregulated compared to the parental strain upon growth on cellulose. (XLS 58 KB)
